# HERCing: Structural and Functional Relevance of the Large HERC Ubiquitin Ligases

**DOI:** 10.3389/fphys.2019.01014

**Published:** 2019-08-07

**Authors:** Jesús García-Cano, Arturo Martinez-Martinez, Joan Sala-Gaston, Leonardo Pedrazza, Jose Luis Rosa

**Affiliations:** Ubiquitylation and Cell Signalling Lab, IDIBELL, Departament de Ciències Fisiològiques, Universitat de Barcelona, Barcelona, Spain

**Keywords:** ubiquitin, ligase, HERC, structure, function, cancer, neurobiology, evolution

## Abstract

Homologous to the E6AP carboxyl terminus (HECT) and regulator of chromosome condensation 1 (RCC1)-like domain-containing proteins (HERCs) belong to the superfamily of ubiquitin ligases. HERC proteins are divided into two subfamilies, Large and Small HERCs. Despite their similarities in terms of both structure and domains, these subfamilies are evolutionarily very distant and result from a convergence phenomenon rather than from a common origin. Large HERC genes, *HERC1* and *HERC2*, are present in most metazoan taxa. They encode very large proteins (approximately 5,000 amino acid residues in a single polypeptide chain) that contain more than one RCC1-like domain as a structural characteristic. Accumulating evidences show that these unusually large proteins play key roles in a wide range of cellular functions which include neurodevelopment, DNA damage repair, and cell proliferation. To better understand the origin, evolution, and function of the Large HERC family, this minireview provides with an integrated overview of their structure and function and details their physiological implications. This study also highlights and discusses how dysregulation of these proteins is associated with severe human diseases such as neurological disorders and cancer.

## Introduction

Proteins containing a HECT domain are ubiquitin ligases (E3). These enzymes participate in the ubiquitylation process accepting ubiquitin from a ubiquitin-conjugating enzymes (E2) and catalysing its transfer to the protein to be ubiquitylated (Buetow and Huang, 2016). In animals, HECT E3 ligases can be divided into 16 groups including the Large HERC family (Marín, 2010), which is the subject of the present minireview. This family is comprised by HERC1 and HERC2, two gigantic proteins of close to 5,000 amino acid residues in a single polypeptide chain. They are the largest HECT-containing proteins^1^.

## Large HERCs Evolutionary Insights

Although traditionally classified together with the Small HERC proteins, Large and Small HERCs form two distant protein families (Marín, 2010). Large HERCs contain more than one RCC1-like domains (RLDs), differing from Small HERCs, which carry only one. Structural differences were observed between the RLDs in Large and Small HERCs (Hadjebi et al., 2008). The explanation for the differences among these two protein groups is that they result from convergent evolution of ancestors belonging to distant families (Marín, 2010).

**Figure 1:**
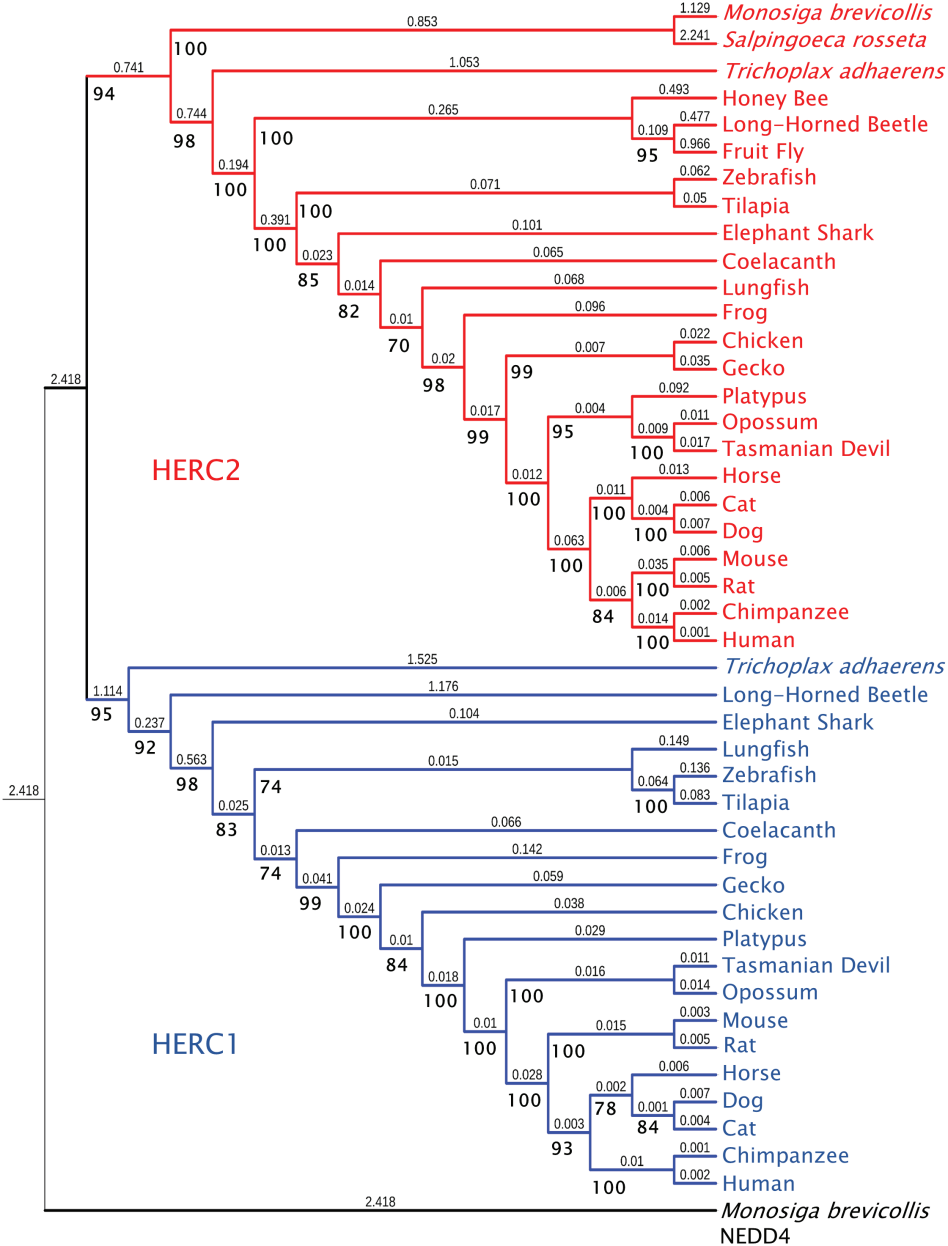
Molecular phylogeny of Large HERC proteins. The tree shows the clustering of HERC1 (blue) and HERC2 (red) amino acid sequences from significative choanoflagellate and metazoan species. Ultrafast bootstrap support (1,000 replicates) is shown next to the branches in bold. Smaller numbers indicate branch lengths. NEDD4 protein from *Monosiga brevicollis* was used as a outgroup.

**Figure 2:**
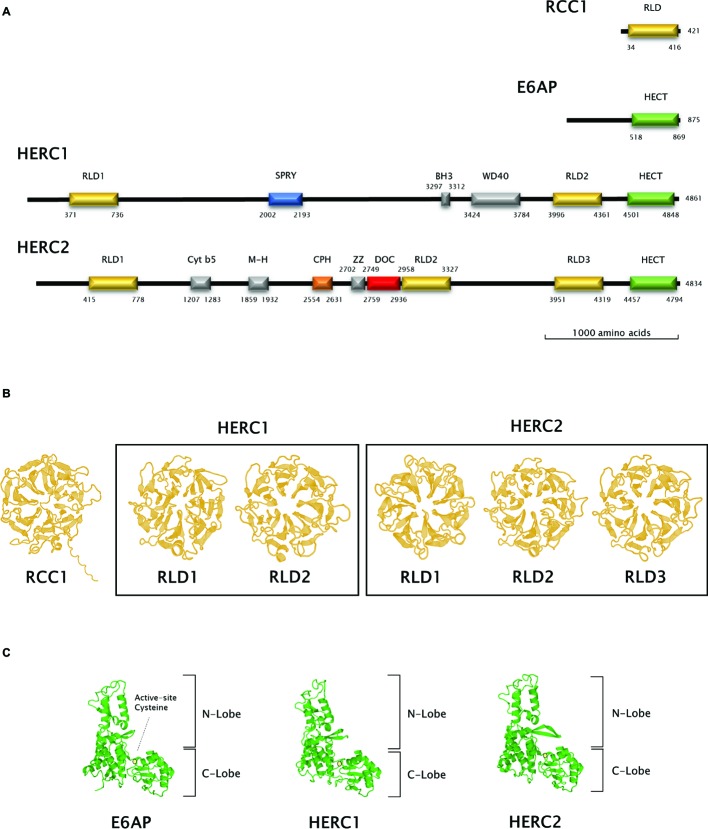
Structural features of Large HERC proteins. **(A)** Primary structure of human RCC-1, E6AP, HERC1, and HERC2 proteins. Scheme shows the approximate relative position of each significative protein domain as indicated by ScanProSite domain predictions with the amino acid sequences. **(B,C)** Three-dimensional structures of homologous RCC1-like and HECT domains in HERC1 and HERC2. Three-dimensional structures of RCC1 and E6AP HECT domains were modelled using the Swiss-Model online software and used as a template for HERC1 and HERC2 RCC1-like **(B)** and HECT **(C)** domains modelling. Active-site cysteine location in the HECT domains is indicated by a pale yellow circle.

While HERC2 appears in some choanoflagellates such as *Monosiga brevicollis* and *Salpingoeca rosetta*, the emergence of HERC1 occurred in Metazoa. Both proteins are already present in the placozoan *Trichoplax adhaerens* and in most metazoan phyla, with the absence of HERC1 in certain insect clades (Marín, 2010). Phylogenetic analysis of Large HERCs amino acid sequences segregates them in two clusters: one for HERC1 and one for HERC2; displaying higher similarity between orthologues (Figure 1). The phylogenetic relationships of the sequences within each cluster correlate with those in the evolution of the species. It is noteworthy that HERC2 from *S. rosetta* presents a SPRY domain, which is characteristic of HERC1 (Figure 2A; Garcia-Gonzalo et al., 2005). However, it cannot be considered homologous to that of *T. adhaerens* HERC1 [19.6% identity, *e* = 0.17; as shown by BLAST-p comparison (Altschul et al., 1997, 2005)]. Thus, this presence is likely due to convergence, a relatively frequent event in HECT proteins along evolution (Marín, 2010).

## Structural Features of Large HERCs

### The RCC1-Like Domains, Structure, and Function

The presence of RLDs is a structural feature of Large HERCs (Figure 2A). RCC1 is necessary for maintaining chromosomes decondensed during DNA replication. It is also a guanine exchange factor (GEF) for the GTPase Ran, a nuclear import protein (Nishimoto et al., 1978; Bischoff and Ponstingl, 1991). RCC1 tertiary structure is composed of seven *β* blades resembling the shape of a propeller (Figure 2B; first panel). Structure prediction models have been used since three-dimensional structure determination of Large HERCs has not been possible to date (Waterhouse et al., 2018). Large HERC RLDs structure is very similar to that of RCC1 (Figure 2B; framed panels).

HERC1 is implicated in intracellular vesicle trafficking by interacting through its RLD2 with ARF1 and Clathrin (Rosa et al., 1996; Rosa and Barbacid, 1997). HERC1 RLD1 may also function as a GDP releasing factor (GRF) for ARF proteins in the presence of phosphatidylinositol-4,5-bisphosphate (Garcia-Gonzalo et al., 2004, 2005). As a small GTPase regulator, HERC1 interacts, among others, with IQGAP1, which is a key interactor centre for such proteins (Jacquemet and Humphries, 2013). No GEF or GRF activities have been reported in HERC2. Of note, HERC2 forms a complex with and stimulates the E6AP ubiquitin ligase activity through its RLD2 (Kuhnle et al., 2011).

### HECT Domain Structure and Function: Ubiquitin Ligase Activity

Large HERCs display a HECT domain at their carboxyl end. *In silico* predicted models show structural similarity with the HECT domain of E6AP (Figure 2C). They form a bilobed structure consisting of a helix-turn-helix motif packed with two and four antiparallel *β* sheets at the N and C ends, respectively. The lobes are joined to the hinge formed by a core of α helices. This bilobed structure facilitates transmission of the ubiquitin residue to its target protein. Thus, the N-terminal-facing lobe is able to bind the E2 enzyme from which the ubiquityl residue is transferred to the catalytic cysteine within the C-terminal-facing lobe of the domain (Figure 2C; circled). Following that, the ubiquitin is transferred to a lysyl residue or to the amino terminus in the target protein (Metzger et al., 2012; Streich and Lima, 2014).

Large HERCs play a role in protein stability. HERC1 regulates C-RAF stability through ubiquitylation leading to proteasomal degradation (Schneider et al., 2018) and is also implicated in the stability of TSC2/tuberin (Chong-Kopera et al., 2006). HERC2 ubiquitylates for proteasomal degradation proteins involved in DNA repair such as XPA and BRCA1, Ubiquitin Specific Proteases (USP) such as USP33 and USP20, and proteins involved in iron metabolism such as FBXl5 and NCOA4 (reviewed in Sánchez-Tena et al., 2016). HERC2 also promotes degradation of the LKB1 kinase when acetylated (Bai et al., 2016).

## Physiological Implications

### Cancer and DNA Damage Repair

HERC2 is implicated in different types of cancer. In osteosarcoma, the increase of HERC2-binding protein SOX18 enhances cell proliferation correlating with a decrease in *HERCs* mRNA levels, especially those of *HERC2* (Zhu et al., 2018). Certain *HERC2* genetic variants are risk factors in cutaneous and uveal melanomas (Ibarrola-Villava et al., 2010; Amos et al., 2011; Kosiniak-Kamysz et al., 2014). Frameshift mutations in *HERC2* have been described in gastric and colorectal carcinomas with microsatellite instability (Yoo et al., 2011).

HERC2 is also implicated in DNA damage repair (DDR). HERC2 induces BRCA1 degradation in breast cancer. This is inhibited either by binding of TUSC4 to HERC2 (Peng et al., 2015) or by BARD1 binding BRCA1 itself (Wu et al., 2010). Moreover, HERC2 targets XPA for degradation. ATR phosphorylates XPA thus preventing this ubiquitylation while WIP dephosphorylates XPA in a circadian manner (Kang et al., 2010, 2011; Lee et al., 2014). Besides, ATR phosphorylates and also stabilizes USP20 by unbinding it from HERC2. In turn, USP20 stabilizes Claspin, which increases the activity of the ATR-Chk1 axis (Yuan et al., 2014; Zhu et al., 2014). On the other hand, HERC2 binds Claspin among other proteins upon MCM2 phosphorylation, and facilitates replication origin firing (Izawa et al., 2011). In a different way, HERC2 promotes resistance to cisplatin and UV-induced DNA damage by stabilizing the Ubc13-RNF8 tandem and RNF168 and enhancing the recruitment of repair factors such as 53BP1, RAP80, and BRCA1 to the damaged sites (Bekker-Jensen et al., 2010; Danielsen et al., 2012; Bonanno et al., 2016; Mohiuddin et al., 2016). Ubc13-RNF8 binding is facilitated by HERC2 by interacting with the RNF8 FHA domain upon phosphorylation of threonine 4827 of HERC2 triggered by ionizing radiation (IR) (Bekker-Jensen et al., 2010) and by acting as a docking platform upon DNA damage, when HERC2 is SUMOylated by PIAS4 (Bekker-Jensen and Mailand, 2010; Danielsen et al., 2012). Both RNF8 and RNF168 are required for ubiquitin foci formation upon IR (Oestergaard et al., 2012), which are necessary for DDR maintenance. In addition to that, HERC2 stabilizes USP16 which, in turn, deubiquitylates histone 2A so as to terminate the DDR (Zhang et al., 2014). Moreover, during S phase, HERC2 is necessary for RPA ubiquitylation which plays a role in clearing G-quadruplex DNA structures by binding the RecQ DNA helicases BLM and WRN (Wu et al., 2018). Finally, HERC2 along with NEURL4 controls cell proliferation by regulating the transcriptional activity of the tumour suppressor protein p53 through regulation of its oligomerization (Cubillos-Rojas et al., 2014, 2017). All these data suggest that HERC2 functions to control the cell response to genotoxic insults and helps maintain genome stability.

HERC1 is also involved in DDR and cancer. *HERC1* deletions affect MSH2 protein levels (Diouf et al., 2011). Besides, it also promotes degradation of BAK in the presence of the E6 protein from the HPV5β virus and prevents cell death in response to UV (Holloway et al., 2015). Furthermore, *HERC1* is recurrently mutated in metastatic triple negative (Craig et al., 2013) and in invasive lobular cancer in the breast (Ping et al., 2016). In addition to that, an atypical *HERC1*-*PML* transcript fusion mRNA (Walz et al., 2010) and HERC1 mutations are also found in leukaemia (Diouf et al., 2011; Neumann et al., 2015; Johansson et al., 2018). HERC1 binds to the M2 isoform of pyruvate kinase, which is typically found in proliferating tissues and cancer cells although the physiological role for this association has not been elucidated to date (Garcia-Gonzalo et al., 2003; Mazurek, 2008). Moreover, as stated above, HERC1 regulates cell proliferation through C-RAF stability. In particular, accumulation of C-RAF upon *HERC1* knockdown results in increased cell proliferation (Schneider et al., 2018). Finally, one singular clinical case of pulmonary sclerosing pneumocytoma revealed several somatic mutations on *HERC1* along with other genes such as *TSC1* and *AKT1* (Fan et al., 2018).

### Development and Neurobiology

In mice, recessive mutations of the *Herc2* gene are associated with defects in growth, motor coordination and fertility (Lehman et al., 1998; Walkowicz et al., 1999). While *Herc2* knockout animals are not viable, heterozygous mice have motor impairment (Cubillos-Rojas et al., 2016). In humans, the *HERC2* gene locates to chromosome 15 among genes responsible for such disorders as Angelman and Prader-Willi syndromes and Autism spectrum disorders (Ji et al., 1999; Dimitropoulos and Schultz, 2007; Roberts et al., 2014). Recessive mutations in the *HERC2* locus are related to symptoms ranging from cognitive delay, ataxia, speech disorders, microcephalia, seizures, facial dysmorphism, hypopigmentation, and other secondary signs such as infections and behavioural alterations (Puffenberger et al., 2012; Harlalka et al., 2013; Neubert et al., 2013; Han et al., 2016; Morice-Picard et al., 2016).

Little is known about the precise molecular mechanism by which HERC2 affects neuronal development and function. HERC2-NEURL4 complex binds RNF8 in neurons regulating synapse formation *in vivo*. Knockdown of HERC2 resembles the effects in RNF8 depletion and inhibition of RNF8-Ubc13 signalling – an increase in parallel fibres, synaptic boutons, and synapse formation with Purkinje cells (Valnegri et al., 2017). HERC2 could also be linked to other neurological diseases. Parkinson’s disease-associated kinase LRRK2 is known to bind to the HERC2-NEURL4 complex thus regulating endosomal vesicular trafficking and promoting the internalization of the Delta-like 1/Delta Notch ligand, affecting its signalling (Imai et al., 2015).

HERC1 has also major implications in neurodevelopment. Herc1 Gly483Glu mutant mice, termed *tambaleante*, present with delayed growth, short body, high juvenile mortality, and severe ataxia, the latter due to Purkinje cell loss beyond the age of 2 months, correlating with autophagy and decreased mTOR signalling (Mashimo et al., 2009). *Tambaleante* mice have impaired neurotransmitter release in neuromuscular junctions (Bachiller et al., 2015), defective ubiquitin-proteasome-driven protein aggregate clearance, and increased autophagic flux in neocortical pyramidal, CA3 hippocampal pyramidal and spinal cord motor neurons (Ruiz et al., 2016) and poor myelinisation and delayed action potential transmission (Bachiller et al., 2018). Furthermore, they have impaired associative learning (Perez-Villegas et al., 2018). In humans, patients with recessive mutations of *HERC1* present with intellectual disability, macrocephalia, facial dysmorphism, feeding difficulties, kyphoscoliosis, thicker corpus callosum, seizures, and other clinical signs resembling autism (Ortega-Recalde et al., 2015; Aggarwal et al., 2016; Hashimoto et al., 2016; Nguyen et al., 2016; Kannan et al., 2017; Utine et al., 2017).

### Other Processes

The human *HERC2* gene locus is upstream of that of the *OCA2* gene (mutated in oculocutaneous albinism) and certain *HERC2* SNPs can interfere *OCA2*’s expression thus affecting eye, skin, and hair pigmentation (Eiberg et al., 2008; Kayser et al., 2008; Sturm et al., 2008; Branicki et al., 2009; Nan et al., 2009). Certain phenotypes are favoured by evolution with a traceable population gradient from Europe to Asia (Ulivi et al., 2013; Wilde et al., 2014). Moreover, polymorphisms in the *HERC2* locus relate to rosacea (Aponte et al., 2018), macular degeneration (Klein et al., 2014), vitiligo (Jin et al., 2012), and skin photosensitivity (Hernando et al., 2018). As for *HERC1*, some of its catalogued polymorphisms are more likely to occur in the East Asian population than in the rest of the world (Xue et al., 2009; Yuasa et al., 2009).

HERC2 is also related to inflammation and autoimmune diseases such as Crohn’s disease, ulcerative colitis, type 1 diabetes mellitus, or sarcoidosis (Franke et al., 2008; Weersma et al., 2009; Wang et al., 2010; Fischer et al., 2011; Garcia-Heredia and Carnero, 2017). With regard to HERC1, a downregulation of its expression has been observed in an LPS-induced inflammation model following coenzyme Q_10_ treatment. (Schmelzer and Doring, 2010).

In addition to this, HERC2 is involved in iron metabolism and ferritinophagy by regulating stability of FBXL5, IRP2, and NCOA4 proteins (Moroishi et al., 2011, 2014; Galligan et al., 2015; Mancias et al., 2015; Ryu et al., 2018) and in toxicology to the response to diisocyanate, toluene, or alcohol intake (Sulovari et al., 2015; Yucesoy et al., 2015; Lim et al., 2017). In regard to toxicology, HERC1 may also play a role in the response of the nervous system to heroin (Drakenberg et al., 2006).

## Final Remarks

Large-HERC family members are staggeringly complex proteins that can intervene in a wide range of physiological processes, such as proliferation, DNA repair, neurodevelopment, inflammation, or ferritinophagy among others. HERC1 and HERC2 sequences are quite conserved through animal evolution, evolving linearly together with the increase of complexity in nervous, endocrine, and immune systems of organisms. Mutations or reduced expression of Large HERCs are associated with neurological disorders, DNA repair defects, and cancer pointing out the importance that Large HERC proteins have in the abovementioned physiological processes.

## Figure Sources

Amino acid sequences were aligned using the Mafft FFT-NS-i algorithm (Katoh, 2002). The phylogenetic tree in Figure 1 was inferred using the maximum likelihood method with IQ-Tree 1.6.9 software (Nguyen et al., 2015). The model used for the analysis was JTT + F + G4, determined using ModelFinder (Kalyaanamoorthy et al., 2017). The IQ-Tree search parameters were set to perturbation strength = 0.8 and 500 unsuccessful iterations to stop (numstop = 500). Ultrafast Bootstrap support (Hoang et al., 2018) was calculated from 1,000 replicates. The final tree obtained with IQ-Tree was visualized using Interactive Tree of Life v4 (Letunic and Bork, 2019). NEDD4 protein from *M. brevicollis* was used as an outgroup. The accession numbers of the sequences used are: for NEDD4: *M. brevicollis* (XP_001750085.1), for HERC1: *T. adhaerens* (XP_002116356.1), *Anoplophora glabripennis* (Asian long-horned beetle) (XP_018567016.1), *Callorhinchus milii* (Elephant shark) (XP_007906088.1), *Latimeria chalumnae* (Coelacanth) (XP_005987767.1), *Danio rerio* (Zebrafish) (XP_021333511.1), *Oreochromis niloticus* (Tilapia) (XP_013121789.1), *Xenopus tropicalis* (Frog) (XP_004916026.1), *Gekko japonicus* (Gecko) (XP_015275623.1), *Gallus gallus* (Chicken) (XP_004943789.1), *Ornithorhynchus anatinus* (Platypus) (XP_028921613), *Monodelphis domestica* (Opossum) (XP_016284147.1), *Sarcophilus harrisii* (Tasmanian devil) (XP_023353554.1), *Equus caballus* (Horse) (XP_023471806.1), *Rattus norvegicus* (Rat) (XP_017451567.1), *Mus musculus* (Mouse) (NP_663592.3), *Homo sapiens* (Human) (NP_003913.3), *Pan troglodytes* (Chimpanzee) (XP_001174017.1), *Canis lupus familiaris* (Dog) (XP_544717.3), *Felis catus* (Cat) (XP_023110993.1), and *Lepidosiren paradoxa* (Lungfish) (Translated from GEHZ01042047.1, NCBI TSA database). For HERC2: *M. brevicollis* (XP_001743304.1), *S. rosetta* (XP_004996155.1), *T. adhaerens* (XP_002112421), Asian long-horned beetle (XP_018562376.1), *Drosophila melanogaster* (Fruit fly) (NP_608388.2), *Apis mellifera* (Honey bee) (XP_01677273), Coelacanth (XP_014343905.1), Zebrafish (XP_021332870.1), Tilapia (XP_013130527.1), Frog (XP_012813156.1), Gecko (XP_015266462.1), Chicken (XP_015133197.1), Platypus (XP_016083014.1), Opossum (XP_007501467.1), Tasmanian devil (XP_023356163.1), Horse (XP_023507879.1), Mouse (NP_001347009.1), Rat (XP_006229362.1), Human (NP_004658.3), Chimpanzee (XP_024204823.1), Dog (XP_022272508.1), Cat (XP_023110801.1), Elephant shark (XP_007889299.1), and Lungfish (Translated from GEHZ01036559.1, NCBI TSA).

Human RCC1 (NCBI accession number P18754) and HECT domain from E6AP (NCBI accession number Q05086.4, amino acids from 518 to 869) three-dimensional structures were modelled using Swiss-Model online software (Waterhouse et al., 2018). RLDs and HECT domains from HERC1 and HERC2 were modelled using a User Template mode. The query sequences were the following: Q15751 for HERC1 (amino acids 371–736 and 3996–4361 for RLD1 and RLD2, respectively, and 4501–4848 for the HECT domain) and O95714 for HERC2 (amino acids 415–778, 2958–3327 and 3951–4319 for RLD1, RLD2, and RLD3, respectively, and 4457–4794 for the HECT domain). PDB files from RCC1 and E6AP HECT domain models obtained as described above were used as templates in these queries. The amino acid position of each domain identified was verified using ScanProSite domain predictions (Sigrist et al., 2012).

## Author Contributions

JG-C and JR conceived and designed the manuscript. JG-C, AM-M, and JR analysed the data and performed figures and tables. JG-C, AM-M, JS-G, LP, and JR wrote the manuscript.

### Conflict of Interest Statement

The authors declare that the research was conducted in the absence of any commercial or financial relationships that could be construed as a potential conflict of interest.

## Footnotes

^1^https://www.uniprot.org/uniprot/?query=reviewed%3Ayes+AND+organism%3A%22Homo+sapiens+%28Human%29+%5B9606%5D%22&sort=mass; (Bateman et al., 2017).
